# Temperature Sensitivity of the Pyloric Neuromuscular System and Its Modulation by Dopamine

**DOI:** 10.1371/journal.pone.0067930

**Published:** 2013-06-28

**Authors:** Jeffrey B. Thuma, Kevin H. Hobbs, Helaine J. Burstein, Natasha S. Seiter, Scott L. Hooper

**Affiliations:** Department of Biological Sciences, Ohio University, Athens, Ohio, United States of America; Goethe University Frankfurt, Germany

## Abstract

We report here the effects of temperature on the p1 neuromuscular system of the stomatogastric system of the lobster (*Panulirus interruptus*). Muscle force generation, in response to both the spontaneously rhythmic *in vitro* pyloric network neural activity and direct, controlled motor nerve stimulation, dramatically decreased as temperature increased, sufficiently that stomach movements would very unlikely be maintained at warm temperatures. However, animals fed in warm tanks showed statistically identical food digestion to those in cold tanks. Applying dopamine, a circulating hormone in crustacea, increased muscle force production at all temperatures and abolished neuromuscular system temperature dependence. Modulation may thus exist not only to increase the diversity of produced behaviors, but also to maintain individual behaviors when environmental conditions (such as temperature) vary.

## Introduction

Ectotherms have lower energy requirements than endotherms because they do not actively maintain a constant body temperature. Taking advantage of this opportunity, at least for ectotherms inhabiting environments with wide temperature variations, requires that life-critical processes function across the temperature range typical of their habitat. Tidal and freshwater crustacea experience wide daily and seasonal temperature changes, and hence would be expected to have evolved temperature compensation mechanisms. Indeed, the firing phase relationships of the pyloric neural network of the crab (*Cancer borealis*) are maintained across a wide temperature range, and modeling suggests this maintenance requires neuron conductances to have similar temperature coefficients [1].

Maintaining normal behavior also requires that neuromuscular function be maintained as temperature varies, but much prior work suggests that crustacean neuromuscular function is strongly temperature-dependent. In crayfish [2], [3], [4] and barnacle [5], warming causes muscle resting membrane potential to hyperpolarize. EJP amplitude, facilitation, and decay time constant vary with temperature in crayfish [2], [3], [4] and crab [6], [7]. In the crayfish *Orconectes limosus*, contraction force in response to muscle depolarization by current injection increases with warming [8], whereas in the crayfish *Astacus leptodactylus* force induced by motor neuron stimulation decreases with warming [2], [3], [4]. These data were mostly obtained in leg muscles. Nonetheless, this temperature sensitivity in other crustacean muscles prompted us to test whether pyloric neuromuscular function, and thus stomach activity, may also not be maintained as animal temperature changes, even if correctly-phased pyloric network rhythmic activity is.

We have performed considerable work on force generation in lobster (*Panulirus interruptus*) pyloric muscles [9], [10], [11], [12], [13], [14], [15], [16]. However, this work did not examine the effects of temperature on neuromuscular function. We show here that pyloric neuromuscular function is highly temperature sensitive, that exogenous dopamine application can overcome this sensitivity, and that dopamine could therefore potentially help maintain muscle function *in vivo*. These data suggest that modulation may function not only to change behavior, but also to maintain it as environmental conditions change.

## Materials and Methods

### Spontaneous Muscle Activity

Spiny lobsters (*Panulirus interruptus*) were obtained from Marinus Scientific (Long Beach, CA) and maintained in aquaria with chilled (9–15°C), circulating, artificial seawater. Stomachs were dissected in the standard manner for muscle preparations [10], [17]. The p1 muscle was freed from the surrounding tissue except for the origin on the lateral ossicle and a small piece of hard connective tissue on the medial end to use as an attachment point for the transducer. The pyloric movement pattern is as yet unknown. However, the p1 muscle is one of the largest pyloric muscles, and the largest of the three muscles innervated by the Lateral Pyloric (LP) neuron. It is thus likely to play an important role in generating pyloric movements. Great care was taken to ensure that muscle innervation remained intact. The muscle was then hooked to a Dual Mode Lever System transducer (Aurora Scientific, Inc) and set to its rest length (approximately 8 mm [16]) by using a ruler and adjusting the height of the micromanipulator to which the transducer was attached. Prior work has shown that pyloric muscles generate substantial contractions in response to motor neuron input at their rest lengths [10], [11], [12], [13], [14], [15], [16]. All experiments were performed in the isometric regime.

During the dissection the preparation was frequently bathed with oxygenated, chilled (∼10°C) *Panulirus* saline (in mM: 479 NaCl, 12.8 KCl, 13.7 CaCl_2_, 3.9 Na_2_SO_4_, 10 MgSO_4_, 10.9 dextrose, 11.1 Tris base, 5.1 maleic acid, pH 7.4–7.6; Fisher Scientific). During the experiment the preparation was continuously perfused with oxygenated (100% oxygen), chilled (9–16°C) saline. The p1 muscle innervation by the LP neuron runs through the lateral ventricular nerve (lvn). A small “window” was cut through the stomach tissue to expose a 10–15 mm length of the lvn just rostral to the p1 muscle. A small piece of Sylgard was placed under the lvn for support and held in place with minutien pins. Extracellular nerve recordings of pyloric network activity were made using stainless steel pin electrodes on the lvn insulated with petroleum jelly and an A-M Systems amplifier.

Saline temperature was changed by varying the length of perfusion tubing immersed in an ice water bath. Perfusion tubing immersion length was varied by the experimenter as necessary to obtain gradual, continuous warming but on the cooling phase the entire length of tubing was typically re-immersed in the ice water bath in a single step. This resulted in the warming phases occurring more slowly than the cooling phases. Saline temperature was monitored using a Physitemp Instruments BAT-12 microprobe thermometer positioned in the dish near the muscle. The p1 muscle is strap-like with an approximate thickness of 1 mm. Given this small thickness and the high temperature conductivity of water, we assume muscle temperature tracked saline temperature with only a small delay.

Temperature, lvn activity, and muscle contraction force were continuously recorded using Spike 2 and a 1401plus (Cambridge Electronic Design). In some preparations, the oxygenated saline was switched to unoxygenated saline, which was normal saline that had been placed in a refrigerator undisturbed for at least 24 hours and then carefully transferred to the perfusion feed flask so as to introduce as little turbulence, and hence atmospheric oxygen, as possible. Oxygen concentrations of the perfusing saline were measured in the dish using a Clarke-type oxygen electrode and a Strathkelvin Model 781 oxygen meter.

### Stimulated Muscle Activity

Dissection was performed as above but the lvn was cut immediately distal to its branch point from the dorsal ventricular nerve (which connects the lvn to the stomatogastric ganglion) and pinned out in a Sylgard dish, taking care to maintain p1 muscle innervation. The muscle was then prepared as described above. Bipolar stainless steel pin electrodes were placed on the lvn and insulated from the bath using petroleum jelly. The lvn was stimulated using the 1401plus and a stimulus isolation unit – 10 spikes at 27 Hz, which is the upper end of the physiological range, chosen to obtain large but still physiologically relevant contractions and with a period long enough to allow the muscle to fully relax between stimulations. Stimulation current was gradually increased until further increases in current did not increase contraction amplitude. The muscle was then allowed to rest for approximately 5 minutes. The muscle was then stimulated as before while saline temperature was altered from 9–22°C. Muscle force was recorded using the same transducer as above and the 1401plus. In some experiments 10^−7^ to 10^−5^ M dopamine (Sigma) containing saline was applied to the preparation.

### Feeding Experiments

Animals were unfed and acclimated to either 9°C or 15°C for at least 48 hours before feeding. Each animal was hand fed 1 g of fish or mussel. The animal was returned to its original tank and left undisturbed for 2 hours, after which the stomach was dissected as above. Undigested food was removed and weighed.

### Data Analysis

As noted above, the slopes of the warming and cooling phases differed. Furthermore, responses to warming and cooling could intrinsically differ. To ensure that all measurements were made under equivalent conditions, quantitative analysis was performed only on contractions in the warming phase of the temperature variations in the lvn stimulation experiments. Measurements were made on continuously changing temperature gradients occurring over ten to fifteen minutes.

LP neuron phase was calculated by measuring the delay from the beginning of each cycle’s PD neuron burst to the beginning of each cycle’s LP neuron burst and dividing by that cycle’s period, defined as the duration from one PD neuron burst beginning to the beginning of the next.

Linear fits and statistical analyses were performed in Origin (OriginLab) and Kaleidagraph (Synergy Software).

## Results

Tang et al. [18] showed that crab (*Cancer borealis*) pyloric network motor neuron output maintained phase across a wide temperature range. We verified that this was also true in lobster in three experiments. Mean pyloric cycle period decreased from 1.38±0.12 s at 9°C to 0.67±0.02 s at 16°C (*p* = 0.009, Student’s paired t-test), a 2.1-fold decrease. Mean LP neuron (the neuron innervating the muscle examined here) phase changed over this temperature range only from 0.42±0.04 to 0.58±0.08. Although this phase change was significant (*p* = 0.023, Student’s paired t-test), it was much smaller than the shift expected (to a phase of approximately 0.9 rather than the observed 0.58) if LP neuron firing delay after PD neuron burst beginning had not shortened as temperature increased. Thus, although not as perfect as that observed in *Cancer*, lobster pyloric networks also showed substantial phase compensation as temperature changed.

**Figure 1:**
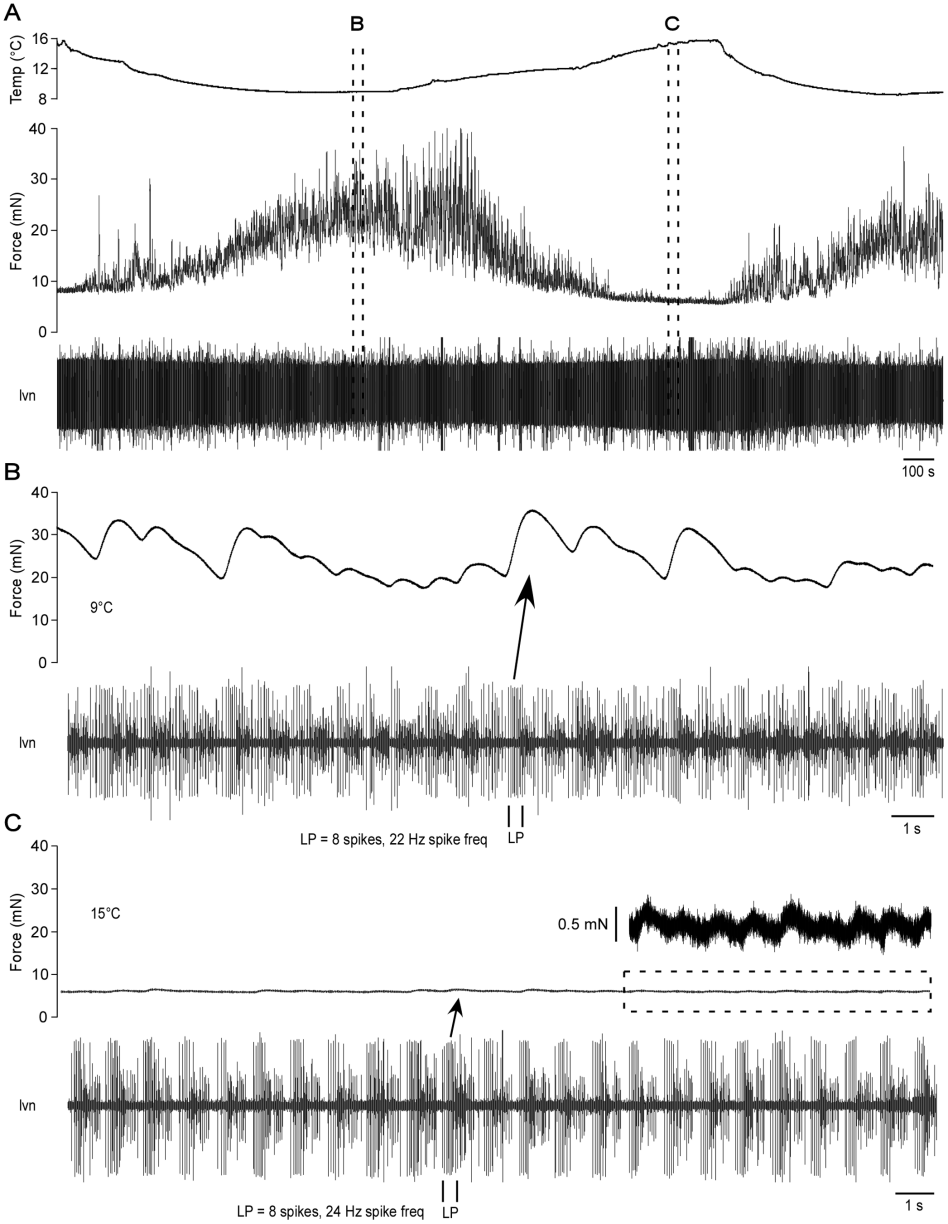
The lobster p1 neuromuscular system is temperature-sensitive. (A) As temperature (top trace) was decreased the force of muscle contraction (middle trace) increased. The activity of the nerve containing the LP neuron input to the muscle is shown in the bottom trace. (B) and (C) Time expansion from (A) showing muscle force (top trace) and neural input (bottom trace) at 9°C and 15°C. Arrows show that two LP neuron bursts chosen for having essentially identical characteristics induced very different muscle contraction amplitudes at the two temperatures. Inset in (C) is muscle force at an expanded scale to show that even at warm temperatures each motor neuron burst continued to induce (very small) muscle contractions.

To test whether this phase-compensated motor neuron output would induce temperature invariant neuromuscular responses, we measured (in animals that had been housed in 9–11°C aquaria) p1 muscle force production in response to spontaneous *in vitro* pyloric network activity as saline temperature was varied (Figure 1). Contraction force was much larger at colder temperatures. In Figure 1A, saline temperature (top trace) was initially approximately 16°C and the muscle showed very small or no contractions (middle trace). As the saline was cooled, the muscle began to contract and muscle contractions continually strengthened as saline temperature decreased. Warming the saline again caused the contractions to weaken and eventually nearly stop. This response was repeatable multiple times within single experiments. Eleven animals were used and in every animal contraction force in warm saline was always much smaller than in cold (0.047±0.046 N at 9°C, 0.007±0.01 N at 16°C, different at *p* = 0.01, Student’s paired t-test), although in some experiments contraction amplitude did not shrink to near zero as in Figure 1A.

Figures 1B and 1C show expansions of small time windows from Figure 1A. Even though the muscle had nearly ceased contracting in Figure 1C, the LP neuron had not stopped firing, as shown by LP neuron action potentials continuing to be recorded at the extracellular electrode (bottom trace, all three panels). Furthermore, at least some of these action potentials further propagated to reach the muscle during every burst, as expansion of the muscle force trace (inset, Fig. 1C) shows that each motor neuron burst continued to induce a (very small) muscle contraction. To make this comparison more explicit, we also identified neuron bursts with nearly identical (each had 8 spikes and 22 or 24 Hz spike frequency) LP motor neuron activity in Figures 1B and 1C. Despite this nearly identical activity at the level of the LP neuron distal axon, the active muscle contraction (the continuous ‘baseline’ force production is due to passive muscle stretch at rest length) at 15°C was essentially zero, even though the 9°C contraction was the largest in the shown data.

**Figure 2:**
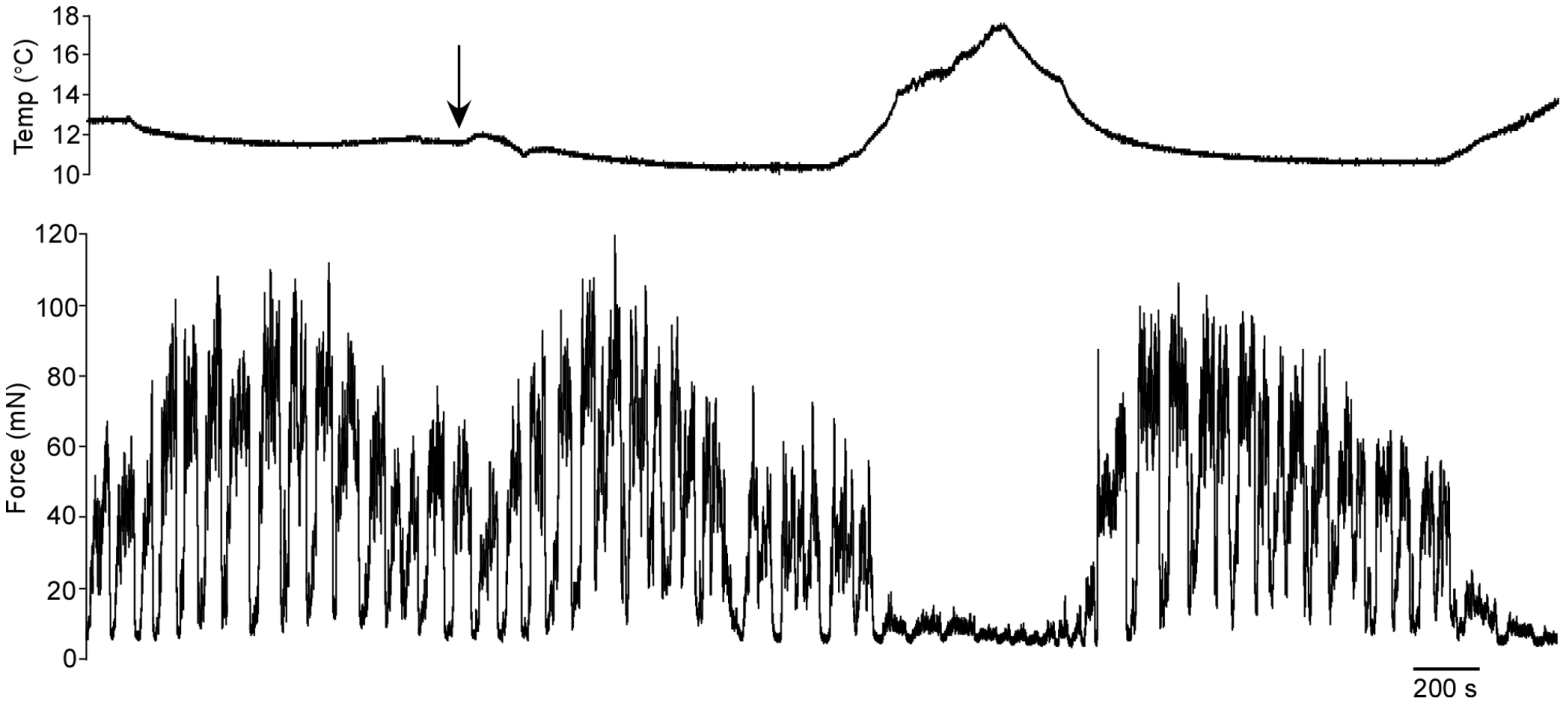
Changing available dissolved oxygen does not affect temperature sensitivity. The preparation was initially perfused with cold, oxygenated saline (top trace) and then switched (arrow) to cold saline containing at least 3 fold less dissolved oxygen. The muscle showed no change in activity (bottom trace) when the reduced oxygen saline was introduced. However, the temperature sensitivity remained and was still reversible despite the much lower oxygen availability. The periodic large decreases in muscle force every 75–100 s are due to the activity of another, much more slowly cycling, stomatogastric network, the gastric mill, which greatly reduces lateral pyloric neuron activity during one phase of gastric mill activity [13], [15].

Gas solubility decreases as temperature increases, therefore decreased oxygen availability could affect neuromuscular function. Although all these experiments were performed in oxygenated saline, it was possible that the temperature dependence of neuromuscular function we had observed was caused by temperature-dependent changes in dissolved oxygen. Three lines of evidence show this possibility is unsupported. First, over these relatively small temperature ranges only very small changes in dissolved oxygen would be expected theoretically [19]. Second, direct measurements showed that dissolved oxygen levels in the dish were essentially identical at 10°C (158 mmHg O_2_) and 15°C (157 mmHg O_2_). Third, we performed experiments (n = 3, animals housed in 9–11°C aquaria) with unoxygenated saline (40 mmHg O_2_ at 10°C, 51 mmHg at 15°C). Thus, this saline contained less than one-third of the oxygen, regardless of temperature, of the oxygenated saline. We repeated the experiment shown in Figure 1, but switched to the unoxgenated saline partway through (Figure 2, arrow). The amount of force the muscle produced did not change when the unoxygenated saline was introduced and the muscle remained sensitive to temperature changes. Temperature-dependent changes in dissolved oxygen thus cannot be causing the changes in neuromuscular function shown in Figure 1.

**Figure 3:**
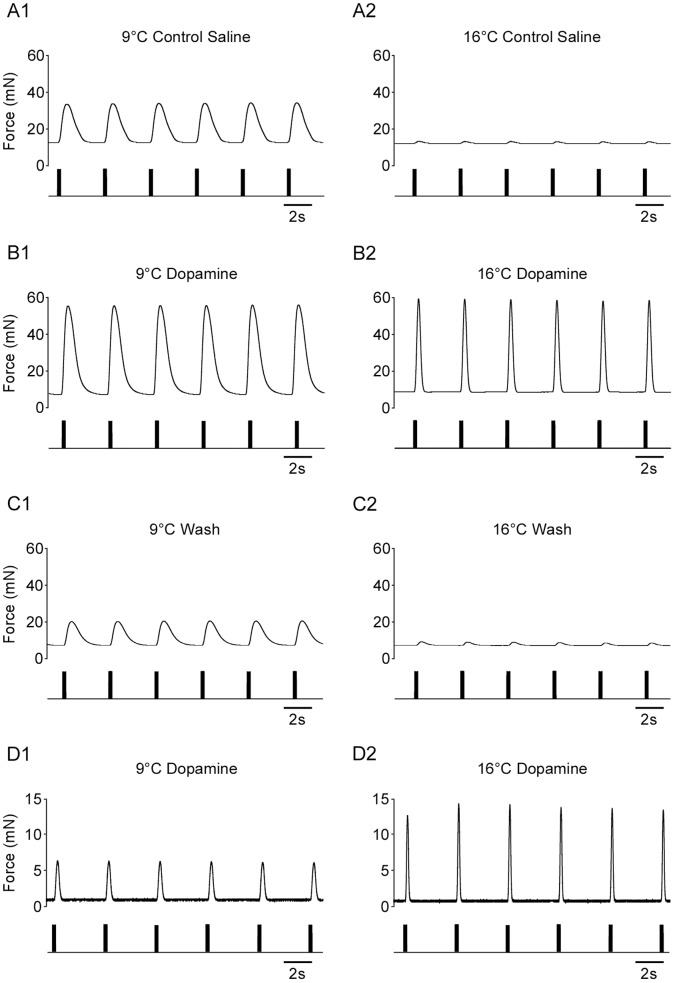
Dopamine blocks temperature sensitivity in an isolated, rhythmically stimulated neuromuscular preparation. (A) A neuromuscular preparation that has been isolated from the spontaneous activity of the pyloric neural network by severing the lvn. Contractions instead elicited by direct motor nerve stimulation with unvarying input (10 spikes at 27 Hz). Muscle contraction amplitude still declined dramatically in warmer saline. This animal had been acclimated to 15°C for 48 hrs, and thus these data also show that neuromuscular temperature sensitivity is independent of animal housing temperature. (B) Adding dopamine (10^−5^) to the saline increased force generation at colder temperatures and allowed the neuromuscular system to continue functioning at warmer temperatures. (C) The dopamine effect reversed upon wash. (D) Dopamine also maintained contraction amplitude in neuromuscular systems from animals held in 9°C aquaria for 48 hrs before the experiment. Data in panels A, B, C all from same animal.

Although pyloric network activity continued at all temperatures, it did change with temperature – in addition to the cycle period and LP neuron phase changes noted above, the LP bursts also became more regular at higher temperatures. In order to ensure that the change in neuromuscular function in response to temperature was not because of these changes in pyloric network activity, we performed experiments in which the lvn connection to the stomatogastric ganglion was cut and we rhythmically stimulated the lvn to provide a completely controlled driving input to the neuromuscular system. Even when receiving this unvarying stimulation, muscle force still strongly decreased in warm saline (Figures 3A1, 3A2). Importantly, and in contrast to the response of the pyloric network to temperature in the crab [18], the animal in Figures 3A1, 3A2 had been acclimated to 15°C for 48 hrs. In 7 out of 8 experiments on animals housed at 15°C the muscles showed much reduced contractions at higher temperatures, including 15°C; 9°C and 15°C contraction amplitudes in these animals (including the one in which contraction amplitude did not decrease with temperature) differed at *p* = 0.018, Student’s paired t-test (0.014±0.009 N at 9°C vs. 0.0035±0.005 N at 15°C. Thus, the sensitivity of the neuromuscular system did not depend on the temperature at which the animals were housed.

These observations raised the question of whether animals housed in warm tanks could digest food. We therefore fed lobsters housed in 9°C and 15°C tanks, returned them to the tanks they had been housed in, and measured how much food remained in their stomachs two hours after feeding. Each animal ingested the full 1 g of food and no difference between the two groups was observed (mean food remaining at 9°C, 0.43±0.12 g; at 15°C, 0.35±0.15 g; *p* = 0.468, Student’s t-test).

The ability of the lobsters to continue to digest food at temperatures at which, *in vitro*, the muscles nearly stop contracting, shows that some compensation mechanism that maintains neuromuscular function must be present *in vivo*. The stomatogastric neuromuscular system is extensively neuromodulated (neural data review [20]; muscle data [21], [22], [23], [24], [25], [26], [27]). We therefore examined the effects of several neuromodulators on neuromuscular function vs. temperature and found that dopamine increased muscle contraction force in cold saline (Figure 3B1, compare to Figure 3A1) and allowed the neuromuscular system to continue to function even in warm saline (Figure 3B2). In dopamine, in which substantial muscle forces were present at all temperatures, it was also possible to observe an additional effect of increased temperature – it resulted in an increased relaxation rate (compare Figures 3B1 and 3B2). This increased relaxation rate would also help maintain the temporal characteristics of muscle contraction with warming (see Discussion). These effects reversed upon wash (Figures 3C1, 3C2). Figures 3A–C are from a single animal housed at 15°C. Whether dopamine had an effect only on animals housed at 15°C was tested by applying dopamine to one animal housed at 9°C; dopamine similarly maintained contraction amplitude at warm experimental temperatures in this animal(Figures 3D1, 3D2).

**Figure 4:**
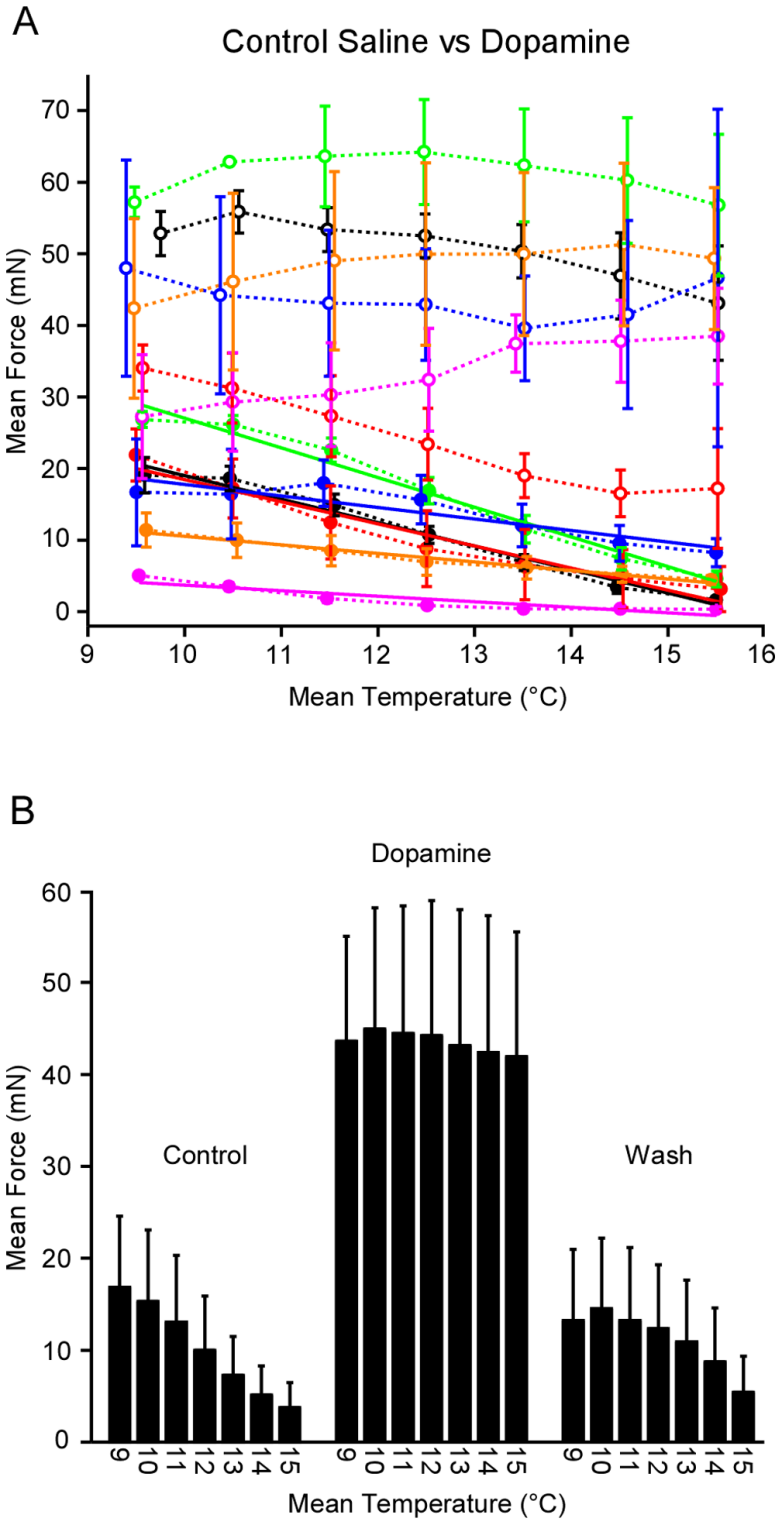
Dopamine allows the neuromuscular system to generate greater force at cold temperatures and continue functioning at warm temperatures. (A) Data from six motor nerve stimulation experiments (closed circles, control saline; open circles, dopamine; matching color lines are data from the same experiment; all animals housed at 15°C). Where present, solid lines show that linear fits to the data were significant. (B) Data for each experiment were binned (1°C) and mean contraction force was determined for each bin. Dopamine differed from control and wash conditions both in amplitude and in temperature sensitivity (present in control and wash, absent in dopamine). See text for statistical details.

Quantitative and grouped data across experiments (all animals housed at 15°C) confirmed the dopamine effects. Figure 4 shows temperature binned (1°C) mean contraction force plots from six lvn-stimulation experiments. Figure 4A shows each experiment’s data (matching colors are data from the same experiment); closed circles are control saline and open circles the dopamine treatment (the wash data are not shown to allow for easier viewing of the control saline fits). At all temperatures, mean contraction force production in dopamine was larger than in control saline. Figure 4B shows across-experiment mean data for each temperature bin. A two-way repeated-measures ANOVA on these data was significant (*p*<0.0001) for both condition (control, dopamine, wash, F = 27.65, 2 degrees of freedom) and temperature (F = 10.46, 6 degrees of freedom). This analysis, however, does not reveal in which condition(s) the temperature dependence is present. We therefore also performed one-way repeated-measures ANOVAs with Tukey’s All Pairs Comparisons on each condition’s data. These showed that control and wash contraction forces depended on temperature at *p*<0.0001 for control (F = 15.87, 6 degrees of freedom) and at *p = *0.004 (F = 4.1, 6 degrees of freedom) for wash, but contraction force in dopamine did not (*p* = 0.91, F = 0.34, 6 degrees of freedom). These analysis thus showed that dopamine not only increased contraction amplitude, but, and more importantly here, removed contraction force’s dependence on temperature. With respect to the nature of the dependence on temperature in control and wash conditions, in control saline the dependence appeared to be linear (solid lines in Figure 4A; all fit *p* values <0.005). In four of the experiments the linear dependence on temperature returned in the wash; in the other two, the linear fits were no longer significant, although contraction amplitude did decrease with increasing temperature.

These experiments were all performed with 10^−5^ M dopamine, a very high concentration for a circulating hormone, and there is no evidence of a direct, local dopaminergic innervation of lobster stomach muscles. We therefore examined the threshold concentration for dopamine’s effects.10^−8^ M and less had no effect on muscle contraction (2 experiments). 10^−7^ M had no effect in three experiments and increased muscle contraction in three experiments, but in only one of these three did contractions continue at warm temperatures (6 experiments total). 10^−6^ M caused larger contractions in three experiments, in two of which the contractions also continued at warm temparatures. The threshold for dopamine effects was thus between 10^−7^ and 10^−6^ M.

## Discussion

We have shown that pyloric neuromuscular function is highly temperature dependent, decreasing to near zero as temperature rises from 9°C to 16°C. This decrease was not because of decreases in dissolved oxygen and was independent of the temperature at which the animals were housed. However, the lobsters continued to digest food at warm temperatures. Application of a known neuromodulator of the stomatogastric system, dopamine, prevented the decrease in muscle contraction force.

### Mechanism Underlying Temperature Dependence

These data give no information as to whether the locus of temperature’s action is pre- or post-synaptic. Pyloric muscles are not innervated at a single end plate, but instead by multiple small processes branching from the main motor neuron axon as it courses over the muscle length, each process ending in one or more synaptic boutons. As such, although the continuance of very small one-for-one contractions with each motor neuron burst even at high temperatures shows that at least some of each burst’s spikes are reaching the muscle, one explanation for the temperature dependence could be increased action potential failure with warming where the small processes branch from the main axon. Warming could alternatively be decreasing the amount of transmitter released per action potential, or any of the postsynaptic steps in the process by which the muscle generates force in response to transmitter binding. Dopamine could similarly work at any level in the neuromuscular system, and in particular need not exert its compensatory effects at the same locus that temperature affects.

### Comparison to Prior Work

This research was undertaken in part because of work showing that the crab pyloric neural network intrinsically maintains phase as temperature increases, which suggested that the pyloric neuromuscular system might show similar intrinsic temperature compensation. We have shown here that it does not, which raises the question of why not. The neural intrinsic compensation results from the electrically-active membrane proteins that underlie pyloric neuron activity changing in a coordinated fashion and to similar degrees with temperature. An argument could be made that neuromuscular system’s failure to compensate lies at the level of the muscles, arising from their requirement to translate electrical activity into force and length changes. This requirement could make them in some way fundamentally more complicated than neurons, which function exclusively in the electrical realm, with this additional complexity preventing intrinsic temperature compensation from evolving in the muscles.

However, from the molecular standpoint no obvious reason exists why it should be more ‘difficult’ for the set of interacting proteins that transform membrane depolarization to actomyosin head rotation to become temperature resistant than it is for the set of interacting proteins that generate pyloric neuron activity. Regardless, despite the functional disadvantages this dependence would entail for such animals unless they live in temperature invariant environments, strong temperature dependence appears to be widely present in crustacean neuromuscular systems (see Introduction). Taken together, these observations suggest that evolving temperature-compensating neuromuscular systems may be more difficult than evolving temperature-compensating neural networks, although why this should be so is unclear.

### Maintenance of Motor Function

In the same manner that most invertebrates can continue to make other movements across wide temperature ranges, our feeding experiments showed that lobster stomachs continue to process food even at temperatures at which, *in vitro*, p1 muscle contractions have essentially zero amplitude. The implied presumption is that modulatory influences (presumably hormonal, as no modulatory neuronal input to pyloric muscles is known) maintains neuromuscular function at elevated temperatures, and dopamine indeed restores neuromuscular function at warm temperatures.

This demonstration does not prove that circulating dopamine in the intact animal serves this purpose. Indeed, the relatively high threshold concentration (between 10^−7^ and 10^−6^ M) for dopamine’s effects suggests that circulating dopamine is unlikely, acting alone, to be responsible for the ability of intact lobsters to digest food at warm temperatures. However, lobsters have large numbers of neuromodulatory substances, many of them hemolymph borne, that increase pyloric neuromuscular function [24], [27]. Dopamine could thus be one of a mixture of circulating hormones that maintains motor function at warm temperatures. More importantly, the demonstration that dopamine can maintain neuromuscular function as temperature changes provides proof of principle that modulators could serve such a role, which was the goal of this portion of this research.

In the presence of dopamine, relaxation rate dramatically increases as temperature increases (compare Figures 3B1 and 3B2). Pyloric muscles relax slowly enough that their contractions summate when driven by physiologically-timed (0.75–2 Hz cycle frequency) motor neuron bursts [11] (the stimulations in Figure 3 were purposefully delivered at a period long enough for full muscle relaxation to occur). Pyloric cycle frequency also strongly increases with increasing temperature in both the lobster (data presented here) and the crab [18]. Even with phase maintenance, this frequency increase, without a change in muscle relaxation rate, would make the muscle contractions become increasingly tonic, displaying only very small rhythmic contractions riding on an underlying large, sustained muscle contraction (e.g., Figure 5 of [11]). As such, if neuromodulators (e.g., dopamine) would allow muscle relaxation rate to increase as temperature increased, such an effect would counteract the temporal summation that would otherwise accompany the increased pyloric rhythm frequency.

### Modulation as a Mechanism to Maintain Behaviors

Modulation is often interpreted as a mechanism for altering existing motor patterns (e.g., changing walk cycle frequency) or creating new ones (walking vs. running). However, another role of neuromodulators, particularly in invertebrates which typically regulate internal conditions less well than vertebrates, may be to maintain existing behaviors as their internal conditions change (temperature, hemolymph osmotic strength, pH, dissolved oxygen levels, etc.). With respect to temperature, such compensation is often believed to be mediated via heat shock proteins [28], [29]. However, the expression and de-expression of these proteins is presumably metabolically more expensive and slower than neuromodulator release and re-uptake. As such, our data suggest it might be worthwhile to examine the relative importance of modulation vs. heat-shock proteins in temperature responses, and also, given the advantages modulation might confer, if temperature-compensating neuromodulators down-regulate heat-shock protein induction.
